# Cytomegalovirus Colitis with Common Variable Immunodeficiency and Crohn's Disease

**DOI:** 10.1155/2015/348204

**Published:** 2015-02-23

**Authors:** Betül Ünal, Cumhur İbrahim Başsorgun, Sinem Çil Gönülcü, Aslı Uçar, Fatih Çelik, Gülsüm Özlem Elpek

**Affiliations:** Department of Pathology, School of Medicine, Akdeniz University, Turkey

## Abstract

Here we present an eleven-year-old male patient who had been diagnosed with common variable immunodeficiency (CVID) three years ago due to recurrent sinopulmonary infections. Two years later he had been diagnosed with Crohn's disease (CD) due to diarrhea episodes which were unresponsive to the treatment. Depending on the active gastrointestinal bleeding and perforation he underwent total colectomy. Despite immunoglobulin and antiviral therapies, general condition of patient deteriorated and he died in the postoperative seventh day. Laboratory analysis was seronegative. CMV inclusion containing cells were detected in postmortem biopsies taken from liver, lungs, and lymph nodes.

## 1. Introduction

CMV colitis is extremely rare in pediatric population. Particularly CMV colitis occurs in immunocompromised patients and rarely in some cases with inflammatory bowel diseases [1, 2]. Primary common variable immunodeficiency disease (CVID) is the second most common in primary immunodeficiency syndromes [1]. The relationship between genetic mutations has been shown, such as TAC-1 and ligands (APRIL, BAFF), which play a role in B cell differentiation and maturation and belong to TNF-like receptor family [3–7]. In addition, defects causing T cell dysfunction were reported in some cases [5, 6]. CVID is associated with recurrent sinopulmonary infections [7]. Although, temporary or persistent diarrhea is frequent complication of disease, CMV infection is rarely seen in gastrointestinal system in patients with CVID [3, 4]. Still in some cases inflammatory bowel diseases can be seen [3–5]. McCurdy et al. [2] investigated risk factors and generated a clinical score to identify patients with inflammatory bowel disease (IBD) at highest risk for cytomegalovirus (CMV) disease. According to their results they reported that patients with medically refractory IBD or endoscopic ulcers and those treated with corticosteroids or immunomodulators, but not tumor necrosis factor (TNF) antagonists, were more likely to have CMV disease than patients with IBD without these features.

## 2. Case Presentation

**Figure 1:**
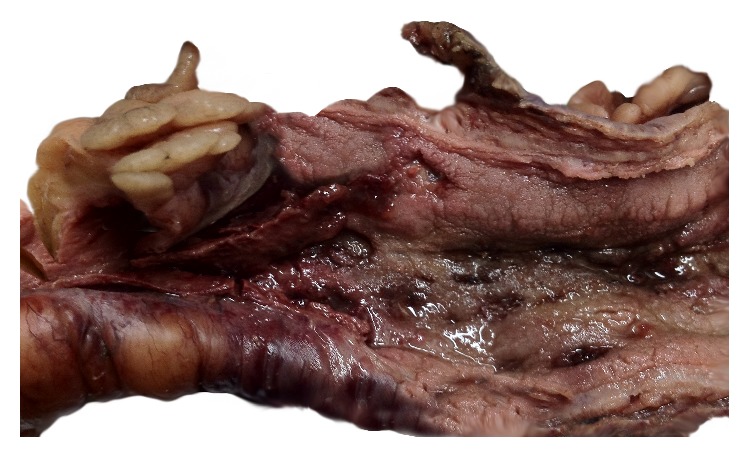
Macroscopic appearance of colectomy material. Mucosa is hemorrhagic and hyperemic; multiple foci of ulcer and perforation areas are seen.

**Figure 2:**
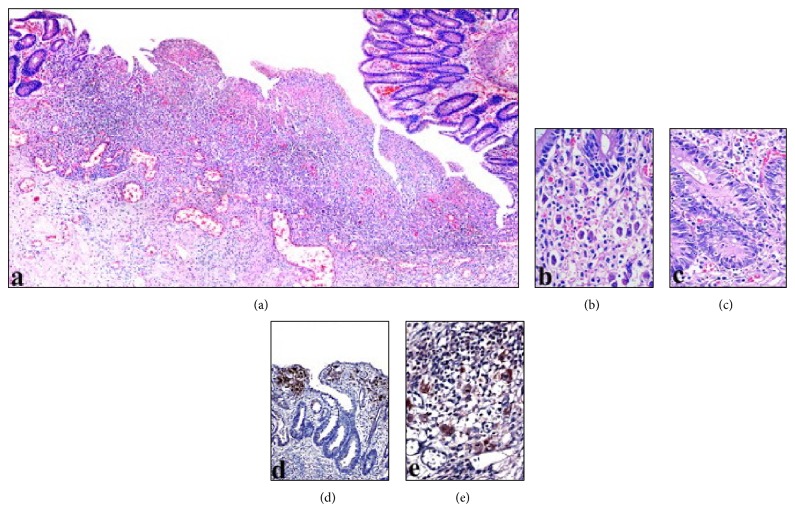
This image shows mucosal ulcer area (a) with the cells containing viral inclusions (b), apoptosis within crypts and PMNL infiltration (c), and immunohistochemical positivity of CMV (d and e) (magnifications (a) ×50; (b, c, and e) ×400; (d) ×200).

**Figure 3:**
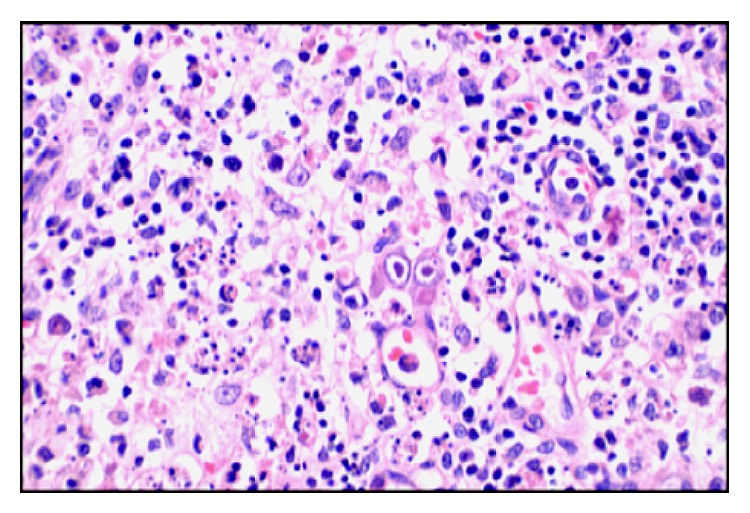
Viral inclusion containing cells seen in lymph node (magnification ×400).

**Figure 4:**
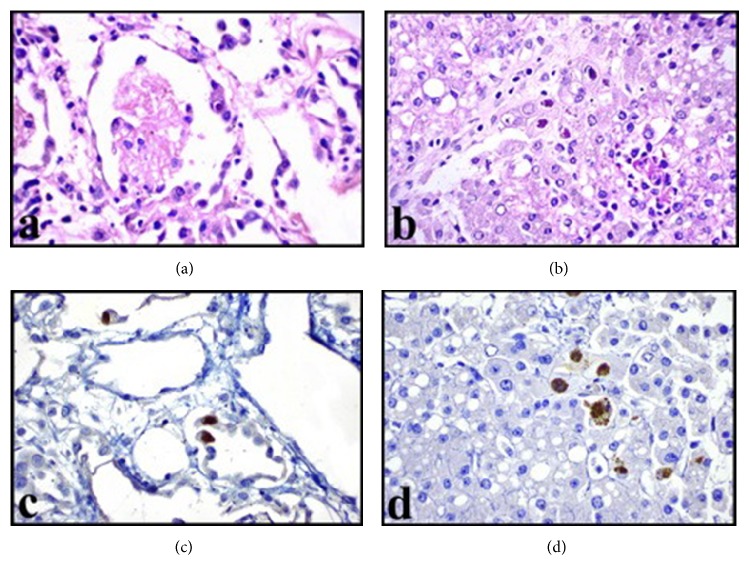
Postmortem biopsies of lung (a) and liver (b) include viral inclusion containing cells. Immunohistochemical CMV positivity in alveolar cells (c) and hepatocytes (d) (magnification (a, b, c, and d) ×400).

Here we present an eleven-year-old male patient who had been diagnosed with CVID three years ago due to recurrent sinopulmonary infections and two years later with CD due to diarrhea episodes which were unresponsive to treatment. He was admitted to our hospital with the complaint of blood in the stool. Laboratory analysis was performed and he was seronegative. He underwent surgery and total colectomy was performed due to active gastrointestinal bleeding and perforation. In macroscopic examination (Figure 1), a perforation area was seen in colectomy material. Mucosa was hemorrhagic and hyperemic, it was flattened in some areas, and multiple foci of ulcer were seen. Microscopic examination revealed intranuclear and cytoplasmic inclusions in fibroblasts, endothelial cells, and mononuclear cells in the base of mucosal ulcer area (Figures 2(a) and 2(b)). In adjacent mucosa, widespread apoptosis within crypts, neutrophilic (PMNL) and eosinophilic infiltration, cryptitis, and a small number of crypt abscesses were seen (Figure 2(c)). Immunohistochemical analysis revealed the CMV positivity in inclusion containing cells (Figures 2(d) and 2(e)). Despite immunoglobulin and antiviral therapies, general condition deteriorated and he died in the postoperative seventh day. CMV inclusion containing cells were detected in postmortem biopsies taken from liver, lungs, and lymph nodes (Figures 3 and 4).

## 3. Discussion

While CMV frequently involves the colon in patients without immunologic disorders, CMV colitis is extremely rare in patients with CVID [3, 4, 6]. CMV colitis carries high mortality risk in CVID and it is more common in organ failure and autoimmune diseases [3, 4]. This condition arises as a result of impaired B cell immunity to be accompanied by impaired T cell response [3, 5]. Apart from that, immunosuppressive therapy of autoimmune diseases such as CD may cause CMV colitis, as seen in our case [3].

Consequently, to prevent mortality and morbidity CMV colitis should be considered in the differential diagnosis of CVID with gastrointestinal complaints. And also this report suggests that immunosuppressive treatment of CD may cause CMV colitis; in addition CMV has the potential to make common diseases. Seronegativity should not mislead the diagnosis; immunohistochemical examination of CMV may be helpful for final diagnosis.
